# The Role of Establishing Neurosurgical Specialist Nurse Working Group in the Recovery and Prevention of Negative Psychological Emotion after Meningioma Surgery

**DOI:** 10.1155/2022/7658710

**Published:** 2022-06-27

**Authors:** Zhen Luo, Qiaoyu Yang, Min Tang, Chaofeng Fan

**Affiliations:** General Ward of Neurology Disease, West China Hospital of Sichuan University, Chengdu, China

## Abstract

In this research paper, we will explore the role of establishing a neurosurgical specialist nurse working group in the recovery and prevention of negative psychological emotions after meningioma surgery. For this study, 42 meningioma patients who were treated before the establishment of a neurosurgery specialist nurse working group from January 2019 to December 2019. They were selected as the control group. In contrast, 42 meningioma patients admitted after the establishment of the neurosurgery specialist nurse group from January 2020 to December 2020 were selected as the study group. The postoperative recovery (time of stay in the intensive care unit, time of first eating, wakeup time, time of defecation for the first time, and hospitalization time), short-term prognosis, and nursing satisfaction scores of the two groups were calculated, and the post-traumatic stress disorder scale (PTSD-SS), medical coping style questionnaire (MCMQ), and National Institutes of Health Stroke Scale (NIHSS) were compared. Also, the changes in the self-rating anxiety scale (SAS) and self-rating depression scale (SDS) score contributes to the comprehensive analysis of the role of the establishment of neurosurgical specialist nurse working group in the recovery and prevention of negative psychological emotion after meningioma operation. The satisfaction scores in the study group of patients in physical care, receiving information, support, respect, and nursing process were higher than the control group probability (*P* < 0.05). The first feeding time, defecation time, out of bed, the stay time in the intensive care unit, and the hospitalization time of the study group were shorter than those of the control group (*P* < 0.05). Before nursing, there was no difference in NIHSS score, SAS score, and SDS scores between the two groups. However, after nursing, the NIHSS score, SAS score, and SDS score of the study group were fairly lower than the control group. Moreover, the Karnofsky functional status scale (KPS) scores of the two groups increased gradually. The KPS scores of the study group at 1 month, 2 months, and 3 months after operation were significantly higher than those of the control group (*P* < 0.05). Before nursing, there was no significant difference in the scores of post-traumatic stress disorder between the two groups (*P* > 0.05). After nursing, the scores of subjective evaluation, symptom avoidance, repeated experience, and social dysfunction in the study group were lower than those in the control group (*P* < 0.05). Before nursing, there was no significant difference in coping scores between the control group and the research group (*P* > 0.05), but after nursing, the avoidance and compliance scores of the research group were significantly higher than those of the control group (*P* < 0.05).Similarly, the scores of avoidance and yield in the study group were significantly lower than those in the control group (*P* < 0.05). In the study group, 1 patient had an incision infection and 1 patient had epilepsy, and the total incidence of postoperative complications was 4.76%. In the control group, 4 patients had incision infection, 1 case of an intracranial hematoma, 3 cases of deep venous thrombosis, and 3 cases of epilepsy. The total incidence of postoperative complications in the study group was 26.19%, while the incidence of postoperative complications in the study group was lower than in the control group (*P* < 0.05).

## 1. Introduction

Meningioma is a tumor in the central nervous system. It originates in the brain or spinal cord. In total, there are three grades in meningioma. It originates from meninges and meningeal spaces and its incidence is high. It accounts for approximately 19% of brain tumors [1]. Meningiomas grow exponentially, and their compression and invasion of brain tissue can lead to a series of adverse consequences, including increased intracranial pressure, local hypoxia, edema, and even brain tissue necrosis [2]. If the tumor is in the skull, then an incision of bone is involved in order to reach the brain. Overall, the procedure is very painful after the surgery. For this purpose, a specialized team of nurses is required to help facilitate the patient after the surgery. Surgical resection is the most effective treatment of meningioma in which mechanical and hypoxic injury is induced by compression and traction during surgery. Inflammatory reaction and oxidative stress caused by the surgery itself can aggravate the craniocerebral injury, and even have a severe effect on the postoperative cognitive function [3–5]. Postoperative intraventricular drainage of meningioma is also an invasive operation, especially when the intraventricular drainage tube is inserted. It promotes bacteria to infect meningioma with drainage fluid reflux. Intracranial infection after meningioma surgery leads to prolonged hospitalization time, increased hospitalization cost, affects the effect of operation and prognosis of patients, and even endangers the life of patients in severe cases. This sometimes results in an increase in mortality of meningioma [6–8]. Therefore, it is of great significance to effectively prevent and control intracranial infection after meningioma operation.

With the deepening of disease research, the influence of negative psychological factors on disease has been paid more and more attention. Negative emotion has become a high-risk factor for the poor prognosis of patients with meningioma after surgery. It plays an important role in all stages of meningioma [9–11]. On one hand, it affects the physiological function of the body, excites the sympathetic nerve, leads to increased heart rate, elevated blood pressure, aggravates cardiac load, and induces or promotes adverse cardiovascular events such as cranial ischemia. On the other hand, it causes social problems and prolongs the average length of stay in the hospital which increases the cost of treatment and family burden and affects the quality of life [12, 13]. The main purpose is to intervene patients after meningioma surgery by establishing a working group of neurosurgery specialist nurses and to observe the effect of the program on the recovery and prevention of negative psychological emotions in patients with meningioma. Adopting this practice can effectively speed up the postoperative recovery process of meningioma patients, reduce the degree of neurological damage, alleviate patients' negative psychological emotions, reduce post-traumatic stress disorder, and improve patients' coping style and prognosis. It plays an optimist role in reducing complication incidence and improving patients' nursing satisfaction.

This research paper is arranged as follows: Section 2 will explain the patients and methods used. It will be followed by results and discussion in Sections 3 and 4. Finally, the research paper is concluded in Section 5.

## 2. Patients and Methods

Following are the clinical information, methods, and statistical analysis used in this research paper.

### 2.1. Clinical Information

Forty-two meningioma patients who were treated before the establishment of neurosurgery specialist nurse working group between the timeline of January 2019 to December 2019. A total of 42 patients with meningioma who received treatment from January 2020 to December 2020 after the establishment of a neurosurgery specialist nurse group in the Department of Neurosurgery were selected as the control group. They were selected as the study group. In the control group, age ranged between 45–65 years. The average age was 57.82 ± 5.62 years old, the body mass was 56–68 kg, and the bodyweight was 63.53 ± 6.43 kg. In the study group, age ranged between 46–68 years. The average age was (64.72 ± 5.89) years, the body mass was 55–70 kg, and the bodyweight was (62.89 ± 6.08) kg.

### 2.2. Inclusion Criteria and Exclusion Criteria

#### 2.2.1. Inclusion Criteria

Inclusion criteria were as follows:

- All patients were diagnosed as meningioma by cranial CT or MRI.
- Surgical resection was smooth and no obvious accident occurred during the operation.
- Normal liver and kidney function and good nutritional status.
- Informed study and signed consent form.

#### 2.2.2. Exclusion Criteria

Exclusion criteria were as follows:

- Patients with previous cerebral hemorrhage, cerebral infarction, and other brain diseases.
- Patients with preoperative mental disorders such as emotional disorders or schizophrenia.
- Pregnancy or lactation.
- Cognitive abnormalities or mental retardation.
- Patients with other serious diseases such as severe intracranial infection.
- Patients with serious complications such as massive hemorrhage after the operation.
- Severe audiovisual impairment affected the correct respondents to the questionnaire.
- Those who had participated in similar research programs.

### 2.3. Methods of Establishing Specialist Nurses Group in Neurosurgery

#### 2.3.1. Set Up a Specialist Quality Research Group

A total of 5 specialist quality research groups were set up, including 2 head nurses, 2 nursing team leaders, and 1 neurosurgical specialist nurse with a bachelor's degree.

#### 2.3.2. Detailed Rules of the Working Group of Specialist Nurses

The group conducted literature inquiry, clinical research, and expert correspondence adjustment and then revised the practice after the trial operation. Finally, they determined the specialist quality indicators and standards. The main results are as follows:

- Through the literature search of “specialist Nursing quality Indexes and Standards” in PubMed, Wanfang Database, China Knowledge Network, and other databases [14], “quality Nursing Service Evaluation rules (2014 Edition)” [15], and hospital nursing system, norms, routines, procedures, as existing quality evaluation standards, as the basis for formulation.
- Through the investigation of the front-line nurses and nursing contents in the department of neurosurgery organized by the research group. The head nurses of various departments and quality control nurses had a group discussion. Based on the principles of importance, maneuverability, sensitivity, representativeness, and specificity, the nursing characteristics of meningioma patients, the admission of surgical patients, high-risk factors, and previous nursing quality problems were analyzed in detail. Select the patient-centered and patient-oriented indicators related to prognosis and outcome that are closely related to nursing quality. At the same time, consider the following factors: take the needs of patients as the nursing purpose, take nursing efficiency as the principle, and pay attention to process quality control.
- Combined with the results of expert letters, it was determined that psychological nursing, infection prevention, and nursing quality were established as sensitive indicators of nursing quality in the neurosurgery department.
- Standardize the above specialist indicators and select seven indicators of organization and management, evaluation, implementation of measures, health education, recording, shift transfer, and related knowledge points to form the above specialist nursing quality standards. Give organizational management, nursing records, health education, shift, and knowledge points, respectively, accounted for 10% of the score weight, and evaluation accounted for 15% of the score weight. The implementation of measures accounts for 35% of the score weight, and the sub-entries of each indicator are given different scores according to the weight. The evaluation includes the first evaluation and dynamic evaluation. The first evaluation should be completed within 2 hours after admission, and the dynamic evaluation should be done once a week at any time when the disease changes. The measures should be implemented to ensure effectiveness, correctness, and individualization. Nursing record standards include recording the results of the first patient evaluation, recording the changes of patients dynamically, recording the corresponding nursing measures, recording the guidance of superior nurses or consultation opinions of professional groups, and recording the effect of evaluation. Nursing records are timely, standardized, accurate, and complete; propaganda and education standards include awareness of existing problems, points for attention in cooperation, and the significance of medical compliance; nursing handover shifts include morning meeting handover, written handover, and bedside handover.

#### 2.3.3. Training, Implementation, and Quality Control

The specialist team first carries on the literature inquiry and clinical data investigation. It is being followed by the trial operation and then the revision. Finally, they determine the specialty quality evaluation index and standard. Specific steps are as follow:

- Through PubMed, Wanfang database, Weipu Medical Network, China Knowledge Network, and other databases about “specialist nursing quality indicators and standards” literature search [14], “quality nursing service evaluation rules (2014 edition) [15], and the hospital nursing system, norms, routines, procedures, and existing quality evaluation standards as the basis for the formulation.
- Through the investigation of the front-line nurses and nursing contents of neurosurgery. It was organized by the research group and discussed in a group by the head nurse of the department and the quality control nurse. Meanwhile, the nursing characteristics of the neurosurgery and meningioma patients, postoperative conditions of patients, high-risk factors of infection, and previous nursing quality problems were analyzed in detail. According to the principles of importance, maneuverability, sensitivity, representativeness, and specificity, patient-centered and patient-oriented indicators related to prognosis and outcome and closely related to nursing quality were selected. In this process, we should take the needs of patients as the nursing purpose, nursing efficiency as the principle, and pay attention to the process quality control.
- Combined with expert opinions and results, psychological nursing, infection prevention, and nursing quality were established as sensitive indicators of nursing quality in the neurosurgery department.
- Standardize the above specialist indicators, and select seven indicators of organization and management, evaluation, implementation of measures, health education, recording, shift transfer, and related knowledge points to form the above specialist nursing quality standards. Give organizational management, nursing records, health education, shift, and knowledge points, respectively. It accounts for 10% of the score weight and evaluation accounts for 15% of the score weight. The implementation of measures accounts for 35% of the score weight and the sub-entries of each indicator are given different scores according to the weight. The evaluation includes the first evaluation and dynamic evaluation. The first evaluation should be completed within 2 hours after admission, and the dynamic evaluation should be done once a week at any time when the disease changes. Measures should be implemented to ensure validity, correctness, and personalization. Nursing record standards include recording the results of the first patient evaluation, dynamically recording patient changes, recording corresponding nursing measures, recording the guidance of superior nurses or consultation opinions of professional groups, and recording evaluation effects. Nursing records are timely, standardized, accurate, and complete; propaganda and education standards include awareness of existing problems, points for attention in cooperation, and the significance of medical compliance; nursing handover shifts include morning meeting handover, written handover, and bedside handover.

#### 2.3.4. Effect Evaluation

A total of 42 meningioma patients were randomly selected before implementation and 42 meningioma patients were randomly selected after implementation (January 2020 to December 2020). The role of neuro-specialist nurses in the recovery and prevention of negative psychological emotion after meningioma operation was evaluated by clinical on-the-spot evaluation, questionnaire survey, and scale evaluation.

### 2.4. Observation Index

The postoperative recovery, short-term prognosis, nursing satisfaction, and complications were compared between the two groups and it is as follows:

1. Postoperative Recovery: It includes the time of stay in intensive care unit, hospitalization time, wakeup time, and the time of defecation for the first time.
2. Short-term Prognosis: The short-term prognosis of the patients was evaluated by the Karnofsky functional status scale (KPS) [16] (1, 2, and 3 months after operation). The score of KPS scale ranges between 0 and 100. The higher the score indicates the better postoperative functional status and prognosis.
3. Complications: It includes incision infection, intracranial hematoma, epilepsy, and deep venous thrombosis.
4. Patients' Nursing Satisfaction: Patients' nursing satisfaction was evaluated with the hospital self-made satisfaction questionnaire. It included five dimensions: physical care, receiving information, support, respect, and nursing flow. Higher score indicates higher satisfaction.
5. Post-traumatic Stress Disorder Scale: The post-traumatic stress disorder scale (PTSD-SS) [17] was used to evaluate post-traumatic stress disorder (PTSD) in patients. The scale consists of 5 dimensions: subjective assessment of traumatic events, avoidance symptoms, recurrent experience, impaired social function, and increased alertness. The score of each item ranges between 1 and 5. Higher score indicates severity of the stress.
6. Medical Coping Style Questionnaire (MCMQ): Medical coping style questionnaire (MCMQ) [18] was used to evaluate the coping style of patients. The scale was composed of three subscales: face (8 items), avoidance (7 items), and submission (5 items). There were 20 items, and the score range of each item was between 1 and 4. Higher score of the face indicates lower score of avoidance yield and positive coping style. The Cronbach's *α* coefficient of each subscale was 0.84–0.90.
7. Self-rating Anxiety Scale (SAS): Self-rating anxiety scale (SAS) [19] was used to evaluate the patients' anxiety. Patients with a total anxiety score of less than 50 were normal, 50/60 was mild, 61/70 was moderate, and those over 70 had severe anxiety.
8. Self-rating Depression Scale (SDS): Self-rating depression scale (SDS) was used to evaluate the depressive parent emotion of the patients. The cut-off value of SDS standard score was 53 points, of which 53–62 points had mild depression, 63–72 points had moderate depression, and more than 73 points had severe depression.
9. Neurological Deficit Scale (NIHSS): The neurological deficit scale (NIHSS) [20] was used to evaluate the recovery of neurological function in patients. Lower score indicates the better recovery of neurological function.

### 2.5. Statistical Analysis

SPSS20.0 statistical software was used for statistical analysis and normal distribution and variance homogeneity analysis were performed on measurement data, which met the requirements of a normal distribution or approximately normal distribution, expressed $\bar{x}\pm s$. The *t*-test was used for comparison between the two groups, and the count data were expressed as *n*(%) and the *χ*^2^ test was used. *P* < 0.05 means the difference is statistically significant.

## 3. Result

### 3.1. Comparison of the Satisfaction Score

The satisfaction scores of patients in the study group in physical care, receiving information, support, respect, and nursing process were significantly higher than those in the control group. The difference was statistically significant (*P* < 0.05). All the dates are presented in Table 1.

### 3.2. Comparison of Postoperative Recovery between the Two Groups

The first feeding time, the first defecation time, the first time out of bed, the stay time in the intensive care unit, and the hospitalization time in the study group was significantly shorter than those in the control group, and the differences were statistically significant (*P* < 0.05). All the dates are presented in Table 2.

### 3.3. Comparison of NIHSS Score, SAS Score, and SDS Score between the Two Groups

Before nursing, there was no significant difference in NIHSS score, SAS score, and SDS score between the control and the study group. However, after nursing, the NIHSS score, SAS score, and SDS score of the study groups decreased lower than in the control group. All the dates are presented in Table 3.

### 3.4. Comparison of Postoperative KPS Score between the Two Groups

The study group and control group were followed up to 3 months after surgery, and 0 cases were lost follow-up. The KPS scores of the two groups increased gradually. The KPS scores of the study group at 1 month, 2 months, and 3 months after the surgery were significantly higher than those of the control group (*P* < 0.05). All the dates are presented in Table 4.

### 3.5. Comparison of Post-Traumatic Stress Disorder (PTSD) between the Two Groups

Before nursing, there was no significant difference in the scores of post-traumatic stress disorder between the control group and the study group (*P* > 0.05). After nursing, the scores of subjective evaluation, symptom avoidance, recurrent experience, social function impairment, and increased alertness were compared between the two groups. The scores of subjective evaluation, symptom avoidance, recurrent experience, impairment of social function, and increased alertness in the study group were lower than those in the control group, and the difference was statistically significant (*P* < 0.05). All the dates are presented in Table 5.

### 3.6. Comparison of Coping Styles between the Two Groups

Before nursing, there was no significant difference in coping scores between the control group and the study group (*P* > 0.05). After nursing, the face scores of the two groups increased. The scores of avoidance and submission in the study group decreased higher than in the control group (*P* < 0.05). The scores of avoidance and yield in the study group were significantly lower than those in the control group (*P* < 0.05). All the dates are presented in Table 6.

### 3.7. Comparison of Postoperative Complications between the Two Groups

In the study group, 1 patient had an incision infection and 1 patient had epilepsy. The total incidence of postoperative complications was 4.76%. In the control group, 4 patients had incision infection, 1 case of an intracranial hematoma, 3 cases of deep venous thrombosis, and 3 cases of epilepsy. The total incidence of postoperative complications in the control group was 26.19%. The incidence of postoperative complications in the study group was significantly lower than in the control group, and the difference was statistically significant (*P* < 0.05). All the dates are presented in Table 7.

## 4. Discussion

Meningioma is a common intracranial tumor derived from arachnoid endothelial cells. It may occur in tissues with arachnoid villi or arachnoid granule components. It accounts for about 16.0–19.2% of intracranial tumors slightly lower than gliomas [1]. Most meningiomas are benign, and some are malignant. 50% of meningiomas occur mainly in the vicinity of the sagittal sinus and other meningiomas such as the convexity of the brain. Falx is also more common, followed by tuberosity sellar, sphenoid ridge, olfactory groove, tentorium, and cerebellopontine angle [21]. It mainly causes local damage to the brain tissue, affects the blood circulation of the brain and blood supply of brain tissue and brings about obstruction of the cerebrospinal fluid circulation pathway. It results in symptoms such as intracranial hydrocephalus, or brain edema, and increased intracranial pressure. Besides, tumor oppression of the optic nerve can cause vision loss, hemi-blindness, loss of vision, and total blindness. It can also cause mental and neurological symptoms. Serious patients can have brain hernias, resulting in their death [22].

The main treatment of meningioma is surgical treatment. Under the premise of ensuring craniocerebral nerve function, total resection of meningioma should be taken as soon as possible. Some patients with malignant meningioma or patients without total resection of meningioma are commonly treated with surgery combined with radiotherapy or chemotherapy [23]. Meningiomas often invade the tissue structures such as blood vessels and venous sinuses at the base of the brain. It is necessary to fully expose the diseased tissue and open the brain tissue during the surgical approach and during a series of treatments such as retraction of brain tissue, surgical resection of the tumor, and postoperative hemostasis [24]. Due to the complexity and long duration of the surgery there are chances of bacterial infection in the intracranial wound and the occurrence of intracranial infection [25]. It also seriously affects the survival rate and quality of life of the patients [23]. Therefore, it is of great significance to strengthen the nursing intervention for the patients undergoing meningioma operation.

Nursing quality management is the core and focus of nursing management. It is an important barrier to ensure the safety of patients and an important means to reflect the value of nursing work [26]. The new round of tertiary hospital evaluation places special emphasis on establishing special nursing quality monitoring indicators. Gradually change from specialized nursing quality monitoring indicators to specialized nursing quality monitoring indicators. With the continuous development of single-disease clinical pathways and nursing methods, medical workers need to formulate a whole-process and specific specialist nursing quality evaluation system in their clinical work [27]. As nurses have the most direct contact with patients, their quality of specialist nursing plays a vital role in the rehabilitation of patients. In the past, the nursing quality evaluation standard system of our hospital was no different than the special standard commonly used in general hospitals. The testing content was mainly basic or terminal quality. They lacked the standards of monitoring and standardization of a specialist quality index. Thus they cannot reflect the characteristics of neurosurgery and lack practical guidance for clinical work [28]. Therefore, it is imperative to perform specialist nursing quality index monitoring and improve the quality of patients' nursing management [29]. Our findings showed that time to first meal, time to first bowel movement, time to first getting out of bed, time in the intensive care unit, and hospital stay were significantly shorter in the study group compared to the control group. It marks that the establishment of a neurosurgery specialist nurse team can significantly speed up the postoperative recovery of meningioma patients and improve patients' satisfaction with the quality of care. Several studies have pointed out that there are some limitations in the domestic hospital visits and health education, such as the narrow scope of visits, lack of postoperative rehabilitation education, lack of professionalism, lack of special interviews and neurosurgery specialties, and lack of evidence-based basis [29]. Some studies have indicated that the incidence of anxiety, depression, and inferiority in patients with postoperative neurological dysfunction is high, which affects the health recovery of patients. However, it can be intervened through psychological guidance to further analyze the effects of the establishment of a neurosurgery specialist nurse team on neurological impairment and negative emotion in patients with meningioma after the surgery. The current results showed that the NIHSS score, SAS score, and SDS score in the study group were lower than in the control group. It is suggested that the construction of a team of specialized nurses in the department of neurosurgery can effectively improve the quality of prognosis and reduce negative emotions.

It is worth mentioning that the postoperative complications and KPS score were important indexes to evaluate the prognosis of patients with intracranial tumors. In this study, the study and control group were followed up to 3 months after the surgery, and 0 cases were lost follow-up. The KPS scores of patients in both groups increased gradually. The scores of the study group at 1 month, 2 months, and 3 months after surgery were higher than those of the control group (*P* < 0.05). It indicates that the construction of a neurosurgery specialist nurse team can reduce the incidence of complications in patients with meningioma and further ensure the quality of prognosis. Based on evidence-based medicine, it is found that the common postoperative complications of meningioma include incision infection, intracranial hematoma, epilepsy, and deep venous thrombosis. However, routine nursing has not formed a complete nursing plan for postoperative complications of meningioma, and the prevention and treatment of perioperative complications is not their top priority. Therefore, the high incidence of complications affects the prognosis. Research shows that preoperative avoidance can protect mental stability to a certain extent, but continued avoidance often has a negative impact on disease [29]. Meanwhile, it causes negative cooperation in the process of perioperative nursing. In the course of the preoperative intervention, this study introduces the positive and negative cases in the previous department and invites optimistic patients to share their own experiences. This gives patients the power and mobilizes their subjective initiative, which is helpful for patients to establish confidence in the surgery and urge patients to face the disease bravely. Wang et al. [30] other studies suggest that craniocerebral injury may exert direct damage to the nervous system and increase the risk of post-traumatic stress disorder. Our present study carries out psychological intervention and gives patients mandala painting therapy after the surgery.

## 5. Conclusion

In light of the above discussion, it can be concluded that the establishment of a neurosurgical specialist nurse working group can provide more scientific and meticulous perioperative nursing for patients with intracranial meningioma. It can help patients integrate the conflict between consciousness and unconsciousness, reduce psychological obstacles, achieve inner order, prevent inner division, and help patients to understand the meaning of life. As the nursing satisfaction of the observation group is higher than that of the control group, it is suggested that the above nursing scheme can improve the nursing satisfaction of patients. It can effectively reduce perioperative complications and promote the recovery of perioperative rehabilitation indexes. It can also improve the short-term prognosis of the patients. However, the effect of this nursing intervention on the long-term prognosis of patient's needs to be conducted in long-term follow-up, and related data can be obtained, which can be further explored in the follow-up study.

## Figures and Tables

**Table 1:** Comparison of patient satisfaction scores between the two groups [*n*/%].

Group	Cases	Body nursing	Accept information	Support	Respect	Nursing process
Control group	42	32.56 ± 4.28	18.66 ± 1.89	14.56 ± 2.52	10.21 ± 2.23	7.97 ± 1.75
Research group	42	41.01 ± 3.14	20.02 ± 2.12	16.53 ± 1.61	12.87 ± 1.35	10.43 ± 0.93
*t*		10.316	3.103	2.269	6.613	8.045
*P*		0.000	0.003	0.000	0.000	0.000

**Table 2:** Comparison of postoperative recovery indexes $\left(\bar{x}\pm s\right)$.

Group	*N*	First feeding time (d)	First defecationtime (d)	Get out of bed for the first time (d)		Length of stay in intensivecare unit (h)	Hospitalizationtime (d)
Control group	42	3.01 ± 1.43	4.18 ± 1.18	3.57 ± 0.76	18.81 ± 2.14		15.36 ± 2.19
Research group	42	1.52 ± 0.83	2.03 ± 1.14	1.62 ± 0.68	15.73 ± 1.84		9.25 ± 1.34
*t*		5.840	8.492	12.392	7.073		15.423
*P*		0.000	0.000	0.000	0.000		0.000

**Table 3:** Comparison of NIHSS score, SAS score and SDS score ($\bar{x}\pm s$, points).

Group	*N*	NIHSS scoring		SAS scoring		SDS scoring	
		Before nursing	After nursing	Before nursing	After nursing	Before nursing	After nursing
Cgroup	42	10.53 ± 1.44	5.98 ± 1.03	57.63 ± 7.38	49.72 ± 5.92	53.84 ± 7.33	45.31 ± 5.68
Rgroup	42	10.57 ± 1.62	5.06 ± 0.83	57.61 ± 7.34	43.22 ± 5.13	53.72 ± 7.36	40.22 ± 4.51
*t*		0.120	4.507	0.012	5.378	0.075	4.548
*P*		0.905	0.000	0.990	0.000	0.941	0.000

Note: the control group before and after nursing, a*P* < 0.05; the study group before and after nursing, b*P* < 0.05.

**Table 4:** Comparison of postoperative KPS scores between the two groups ($\bar{x}\pm s$, points).

Group	*N*	Before operation	One month afteroperation	Two months after operation	Three monthsafter operation
Control group	42	70.21 ± 4.23	75.42 ± 4.67	77.64 ± 4.18	78.04 ± 4.58
Research group	42	71.32 ± 4.61	80.73 ± 5.14	83.84 ± 4.82	85.64 ± 5.69
*t*		1.150	4.955	6.298	6.743
*P*		0.254	0.000	0.000	0.000

**Table 5:** Comparison of post-traumatic stress disorder between the two groups.

Group	*N*	Subjective assessment		Avoid symptoms		Repeat the experience over and over again		Impaired social function		Increased alertness	
		Before nursing	After nursing	Before nursing	After nursing	Before nursing	After nursing	Before nursing	After nursing	Before nursing	After nursing
C group	42	3.01 ± 0.97	2.09 ± 0.54^a^	29.26 ± 3.11	20.34 ± 2.77^a^	21.14 ± 3.91	17.27 ± 3.15^a^	7.03 ± 1.11	5.16 ± 0.83^a^	19.94 ± 3.72	15.83 ± 3.26^a^
R group	42	2.93 ± 1.01	1.28 ± 0.45^b^	28.54 ± 3.66	16.53 ± 2.15^b^	20.64 ± 4.02	14.03 ± 2.45^b^	6.95 ± 1.34	3.26 ± 0.74^b^	20.36 ± 3.18	12.75 ± 3.14^b^
*t*		0.370	7.468	0.972	7.042	0.578	5.262	0.298	11.073	0.556	4.410
*P*		0.712	0.000	0.334	0.000	0.565	0.000	0.767	0.000	0.580	0.000

Note: control group before and after nursing, ^a^*P* < 0.05. Study group before and after nursing, ^b^*P* < 0.05.

**Table 6:** Comparison of coping styles between the two groups.

Group	Face		Avoidance		Yield	
	Before nursing	After nursing	Before nursing	After nursing	Before nursing	After nursing
Control group	19.78 ± 2.63	23.12 ± 3.41	20.19 ± 1.94	16.08 ± 1.34	14.08 ± 2.66	11.34 ± 2.08
Research group	20.14 ± 2.87	26.08 ± 3.16	20.42 ± 2.08	12.04 ± 1.76	14.92 ± 2.57	8.89 ± 1.67
*t*	0.599	4.126	0.524	11.836	1.472	5.592
*P*	0.551	0.000	0.602	0.000	0.145	0.000

**Table 7:** Comparison of postoperative complications between the two groups [*n*/%].

Group	*N*	Incision infection	Intracranial hematoma	Deep venous thrombosis	Epilepsy	Total incidence rate
Control group	42	4 (9.52)	1 (2.38)	3 (7.14)	3 (7.14)	11 (26.19)
Research group	42	1 (2.38)	0 (0.00)	0 (0.00)	1 (2.38)	2 (4.76)
*χ* ^2^						7.372
*P*						0.007

## Data Availability

The datasets used and analyzed during the current study are available from the corresponding author upon reasonable request.
